# A Pharmacokinetic Study of Native *E.coli* Asparaginase for Acute Lymphoblastic Leukemia Treated with ThaiPOG Protocol

**DOI:** 10.31557/APJCP.2021.22.10.3309

**Published:** 2021-10

**Authors:** Phumin Chaweephisal, Trai Tharnpanich, Aphinya Suroengrit, Pattramon Aungbamnet, Panya Seksarn, Darintr Sosothikul, Supanun Lauhasurayotin, Kanhatai Chiengthong, Hansamon Poparn, Piti Techavichit

**Affiliations:** 1 *Clinical Research for Holistic Management in Pediatric Hematology and Oncology, Department of Pediatrics, Faculty of Medicine, King Chulalongkorn Memorial Hospital, Chulalongkorn University, Bangkok, Thailand. *; 2 *Faculty of Pharmaceutical Sciences, Chulalongkorn University, Thailand. *

**Keywords:** Native, asparaginase, silent inactivation, allergy, pharmacokinetic

## Abstract

**Background:**

Asparaginase is one of the essential chemotherapies used to treat acute lymphoblastic leukemia (ALL). Asparaginase antibody production may cause a subtherapeutic level and result in an inferior outcome. The aim of this study was to prove the efficacy of current native *E.coli* asparaginase-based protocol. Moreover, does subtherapeutic result appeared in small group of the trial?.

**Methods:**

A prospective study of asparaginase activity among patients who received native *E.coli* asparaginase 10,000 IU/m^2^ intramuscularly according to The Thai Pediatric Oncology Group (ThaiPOG) protocol was done. The plasma asparaginase activity was measured by the coupled enzymatic reaction. Pharmacokinetic data including peak activity (C_max_), time to maximum concentration (T_max_), area under the curve (AUC_0-48h_) being elucidated.

**Results:**

Eight patients (five males and three females), median age 9.5 years, were enrolled. The median asparaginase activity of seven cases who were eligible for calculation reached Tmax within 24 hours (range 6-48 hours) with mean±SD of C_max_ 3.60±0.34 (range 3.02-4.11) IU/ml. Mean±SD of AUC_0-48h_ is 143.23±36.94 IU.h/mL (range 71.07 – 180.12 IU.h/mL). The post-48-hour activity showed a mean±SD of 3.19±0.24 IU/ml (range 2.77-3.51 IU/ml) which implied an adequacy of activity over 48 hours and proper for the 12-day period. One relapsed ALL patient showed an extremely low AUC of asparaginase activity which coincided with urticaria after asparaginase injection. Subsequently, the asparaginase antibody was demonstrated in this patient.

**Conclusion:**

Native E. coli asparaginase-based protocol provides a compelling pharmacokinetic effect. Asparaginase activity and/or antibody testing is recommended for all cases especially in a relapsed patient, history of high accumulative dose of asparaginase or suspected allergic reaction. Patients with low asparaginase activity or allergy may benefit from switching to an alternative form of asparaginase to maintain treatment efficacy.

## Introduction

Acute lymphoblastic leukemia (ALL) is the most common malignancy in childhood accounting for 22.2 per million person-years between the ages of 0-19 years in Thailand with an age-standardized incidence rate ranging from 1.08 to 2.12 per 100,000 person-years worldwide (Bidwell et al., 2019; Katz et al., 2015). Many factors affect the survival rate including age, induction remission status, cytogenetics, and central nervous system involvement. Recent data have shown that the level of certain chemotherapy agents may affect the survival rate especially asparaginase which is the backbone chemotherapy in many phases of treatment (Wetzler et al., 2007; Riccardi et al., 1981). A study in adult ALL age 17 to 71 revealed that patients who could not maintain plasma asparaginase activity greater than 0.03 IU/ml at post-14 days had inferior overall survival (OS) (p = 0.002; HR = 2.37; 95% CI = 1.38-4.09) and reduced disease-free survival (DFS) (p = 0.012; HR = 2.21; 95% CI = 1.19-4.13) (Wetzler et al., 2007). A study of monkeys and humans demonstrated that following intravenous asparaginase administration, an plasma asparaginase activity of more than 0.1 IU/ml can deplete the central nervous system (CNS) asparagine in both species and may cause effectiveness (Riccardi et al., 1981). The descriptive study of 262 patients received native *E.coli* asparaginase from two different manufacturer Aginasa^®^ and Leuginase^®^ showed post-48 hour activity (above 0.1 IU/ml) achieved in 81% and 3% of patients respectively. The six patients died, five with active disease in the only group of Leuginase^®^. It might be a good demonstration of correlation of activity and outcome (Cecconello et al., 2018).

The main mechanism of asparaginase works to metabolize L-asparagine to L-aspartic acid. The process causes depletion of asparagine which is an essential amino acid for the leukemic cells (Ho et al., 1970). Asparagine deficiency ceases cell differentiation and induces cell death. Nowadays, three or more forms of asparaginase are available, native *E.coli* asparaginase, pegylated asparaginase, and Erwinia asparaginase (Metayer et al.,2019). Native *E.coli* asparaginase is the prototype first introduced in ALL treatment in 1968 and has subsequently shown to increase remission rate from 86 to 93% when combined with other treatments (Ortega et al., 1977). However, pegylated asparaginase has currently replaced native *E.coli* and is used as the first-line drug because of its more rapid clearance of lymphoblasts cells in bone marrow, prolonged plasma asparaginase activity, and lower hypersensitivity events (Ortega et al., 1977; Avramis et al., 2002). If native *E.coli* pegylated asparaginase allergy develops, Erwinia asparaginase is indicated because of its immunological distinction and lack of cross reactivity (Egler et al., 2016).

This study was based on the standard chemotherapy guidelines of Thailand under the ThaiPOG 2018 protocol (The Thai Pediatric Oncology Group, 2018). The classification risk of ALL patients was stratified as standard, high, and very high risk and correlated with the standard, high, and very high-risk ThaiPOG protocols. The guidelines were adapted from the Children’s Oncology Group (COG) guideline (COG AALL0932(Children’s Oncology Group, 2015), AALL1131(Children’s Oncology Group, 2015)). For ThaiPOG 2018, asparaginase was administrated intramuscularly exclusively in the form of native *E.coli* asparaginase. All risk groups completed up to five sessions of asparaginase from induction to delayed intensification (DI) phase. Each session is comprised of 6 doses of 10,000 IU/m^2^ given every other day and completed within 12 days.

One pharmacokinetics study of native *E.coli* asparaginase (6,000 IU/m^2 ^intramuscular administration in the induction phase and DI phases 1 and 2) revealed that peak activity in one patient reached 2 IU/ml at 4 hours after injection (Avramis et al., 2002). The sample of 59 patients had a mean elimination half-life of 1.8 days during the induction phase and 1.5 days in DI 1-2. Adequate plasma asparaginase activity in DI 1-2 determined by activity above 0.1 IU/ml was found in 19-22% of cases after 21 days of administration (Avramis et al., 2002).

Another pharmacokinetics study of native *E.coli* asparaginase, mostly 10,000 IU/m^2^ intramuscularly in some cancer patients, documented that plasma activity could be detected initially within the first hour (Ho et al., 1981). The peak activity occurred between 14 to 24 hours at a level of 1.12±0.14 IU/ml. The half-life was 41.7±4.3 hours (range 34-49 hours). The durable activity lasted above 0.1 IU/ml for more than 8 days. Volume distribution (Vd) was 245 ±28 ml/kg and area under the curve (AUC) was 18 - 22 IU/ml per hr. This study identified one case with a very low peak activity of 0.04 IU/ml. The presumptive explanation might be from obesity which traps the drug subcutaneously and impacts antibody production or other parts of the proteolytic degradation process (Riccardi et al., 1981; Walenciak et al., 2019).

Asparaginase is not normally found in humans, therefore, it is possible to induce the production of autoimmunity against the drug. With the antibody production, some patients may have the clinical appearance as urticaria or anaphylaxis from hypersensitivity type I mechanism. However, other patients do not have any clinical manifestations which is called silent inactivation. The major problem with both types of response groups is that lowering the plasma asparaginase activity can reduce effectiveness. The antibody was found in 28% (16 of 57) of native *E.coli* asparaginase and 12% (7 of 56) of pegylated asparaginase. Both types found cross-reactivity between the groups. Erwinia asparaginase which is produced from fungus of a species of E.chrysanthemi also has hyperreactivity, but does not cross-react with other types (Wang et al., 2003). The antibody-positive status is an indication to shift the drug to another formula to maintain the efficacy of leukemia treatment (Dinndorf et al., 2007). The aim of this study was to prove the efficacy of current native *E.coli* asparaginase-based protocol. Moreover, does subtherapeutic result appeared in small group of the trial?

## Materials and Methods

*Study objectives*

This is the prospective pharmacokinetic study of native *E.coli* asparaginase at 10,000 IU/m^2^ administration intramuscularly during the induction and DI phase following the ThaiPOG 2018 protocol for childhood ALL. The results were documented by measuring plasma asparaginase activity and calculating pharmacokinetic parameter values. The appropriate activity is determined by a level above 0.1 IU/ml throughout the treatment period which indicates drug efficacy. This method emphasizes drug monitoring, which is now routinely practiced in some countries.. The study may also support the effectiveness of the native *E.coli* asparaginase formula used in the ThaiPOG2018 protocol.

*Ethics and inclusion/exclusion criteria*

The study was approved by the Institutional Ethics Committee, King Chulalongkorn Memorial Hospital (IRB number: 258/63). The inclusion criteria were all patients who received ALL protocol between the age of 1 to 18 years, limited to 8 cases. Patients were still under induction (phase I) or DI (phase IV) period at King Chulalongkorn Memorial Hospital (KCMH), Bangkok, Thailand from September 2020 through June 2021. The exclusion criteria included patients less than 1 year, body weight below 10 kilograms or 3^rd^ percentile, and parent refusal to participate. Eight eligible patients were recruited. Native *E.coli* asparaginase, under the tradename of LEUNASE^®^ manufactured by Kyowa Kirin Corporation, Japan, was administrated intramuscularly at a dose of 10,000 IU/m^2^ 6 doses given every other day over a period of 12 days 

*Sample collection and quantification methods*

Blood samples were drawn via a central venous catheter a total of 9 times: baseline (0) before injection, and then at 0.25, 0.5, 1.0, 2.0, 4.0, 6.0, 24, and 48 hours after the first dose, respectively. The post-first dose sample was taken to ensure the activity level would not be affected by previous injections. However, the monitoring was limited to 48 hours after second dose administration. Each sample was filled in a heparin tube and centrifuged at 3,000 rounds per second for 15 minutes to separate the plasma. The products were stored at -80 degrees Celsius. All steps were processed within 1 hour after collection. Time collection was recorded. The plasma asparaginase activity were measured by the coupled enzymatic reaction using Asparaginase Activity Assay Kit (Raybiotech^®^, United States) product. The reaction was determined by using the Varioskan Flash spectral scanning multimode reader at 570 nm (Thermo Scientific^®^, Thailand). The day-to-day variation was controlled by process ELISA at one point-in-time after all samples had been collected.

If detected low asparaginase activity, the plasma asparaginase-specific antibody testing would be performed on post-48 hours of the last of all asparaginase doses. The human asparaginase antibody kit (Mybiosource®, United states), the coupled enzymatic reaction technique, was used for antibody quantitative measurement.

*Statistical analysis*

The baseline activity (0 hour) was utilized for calibration. The pharmacokinetic parameters were calculated by non-compartmental analysis (NCA) model and PK software program (PCModfit® version 6.9, UK). The area under the curve (AUC) was demonstrated by linear trapezoidal rule (AUC_0-inf_) reflecting data from 0 to 48 hours after injection.

## Results

Demographic data are shown in Table 1. Eight patients were recruited, five males and three females. The median age was 9.5 years (range 1-12 years-old). Seven patients who were newly diagnosed received the ThaiPOG ALL protocol which divided the patients into 3 risk categories: standard, high, and very high risk. One patient who was diagnosed with late combined medullary and CNS relapsed disease received the high-risk chemotherapy protocol due to the decision to proceed without hematopoietic stem cell transplantation. All patients followed the ThaiPOG 2018 guidelines requiring native *E.coli* asparaginase 10,000 IU/m^2^ 6 doses given intramuscularly every other day over a period of 12 days and with regular follow up for complications more than six months. All complications were recorded. The concurrent combination chemotherapies are vincristine, doxorubicin, intrathecal methotrexate, and systemic steroid, all identically. Antimicrobial agents and supportive drugs were not considered in our study.

**Figure 1:**
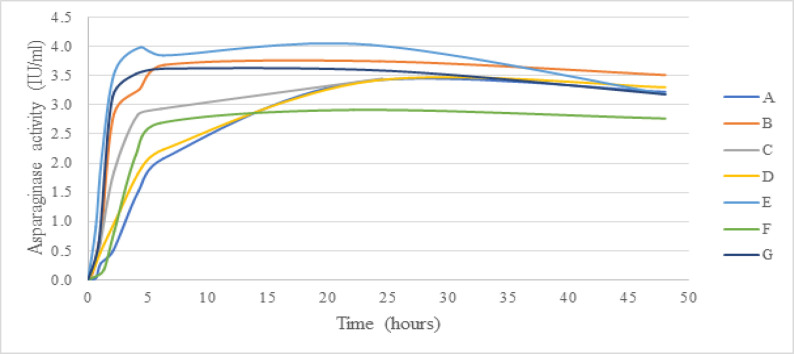
Asparaginase Activity of Seven Cases who are Eligible for Calculation at Selected Time Points after Native *E.coli* Asparaginase Intramuscular 10,000 IU/m^2^ Injection

The asparaginase activities of seven cases were measured at all 9 time points and displayed in Figure 1. The pharmacokinetic parameters were shown in Table 2 (excluding one case (case H) with extremely low values). The time-to-peak activity level (T_max_) appeared most often at 24 hours (range 6-48 hours). The mean maximum concentration (C_max_)±SD was 3.60±0.34 IU/ml (range 3.02-4.11 IU/ml), then decrease subsequently. Mean±SD of AUC_0-48h_ was 143.23±36.94 IU.h/mL (range 71.07 – 180.12 IU.h/mL). The post-48-hour activity mean±SD was 3.19±0.24 IU/ml (range 2.77-3.51 IU/ml) which implied an adequacy of activity over the 48 hours and the total 12 days period for all seven cases. We successfully showed the peak level of the drug, but failed to demonstrate the half-life because of the limitation of the 48-hour interval between each dose. The highest AUC_0-48h_ exhibited was case E. It was presumed most potentially to have some side effect. However, there is no report any side effects of asparaginase including hypercoagulable state, coagulopathy, pancreatitis, and hyperbilirubinemia. Additional data are required to support a more definite correlation.

**Figure 2:**
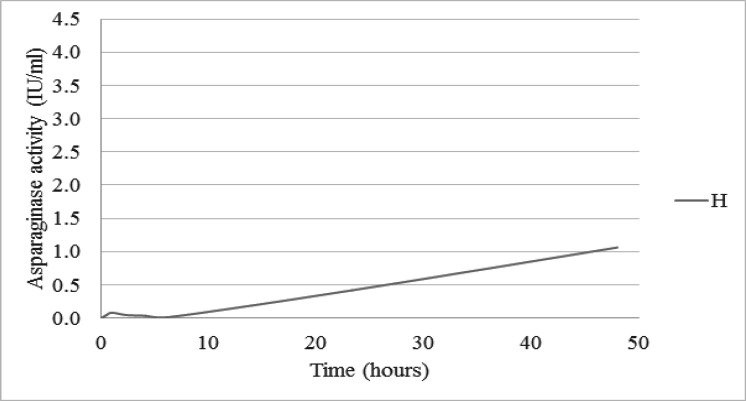
Asparaginase Activity of Another One Case at Selected Time Points after Native *E.coli* Asparaginase Intramuscular 10,000 IU/m^2^ Injection

The extremely low pharmacokinetic values of another one case (H) were shown in FIGURE 2 and Table 3. This patient had a medical history of combined medullary/CNS relapse status which coincided with clinical allergy as urticaria for the last two doses of native *E.coli* asparaginase on DI time. Insufficient data prevents an explanation of whether a lower AUC might be a factor for disease relapse. Further investigation of case H demonstrated an asparaginase-specific antibody-positive status (Table 3). 

**Table 1:** Demographic and Clinical Data of Patients (n=8)

Data	N (percent)
Diagnosis	
ALL	7 (87.5%)
T-lymphoblastic lymphoma	1 (12.5%)
Phase of study	
Induction	3 (37.5%)
Delay intensification	5 (62.5%)
Age (years)	
0 - 3	2 (25%)
4 - 10	2 (25%)
10 - 13	4 (50%)
> 14	0
Initial WBC count (cell/mm^3^)	
< 10,000	6 (75%)
10,000-50,000	0
> 50,000	2 (25%)
CNS involvement ^¥^	
Yes	2 (25%)
No	6 (75%)
Cytogenetics	
Normal	5 (62.5%)
Abnormal^ ±^	2 (25%)
No data	1 (12.5%)
Remission after 28 days of induction	
CR1	7 (87.5%)
CR2	1 (12.5%)
MRD status after 28 days of induction	
Positive	0
Negative	8 (100%)
Chemotherapy ALL protocol (corresponding to risk stratification)	
Standard	1 (12.5%)
High^π^	6 (75%)
Very high	1 (12.5%)
Relapse after 6 months enrollment	0
Complication (exclude infection)	
Asparaginase allergy (urticaria)	1/8
Methotrexate allergy (maculopapular rash)	1/8
Bactrim allergy (anaphylaxis)	1/8
Fracture tibia	1/8

Abbreviation: ALL, acute lymphoblastic leukemia; CNS, central nervous system; CR1, first complete remission; CR2, second complete remission; MRD, minimal residual disease; WBC, white blood cells; ^¥ ^CNS involvement defined by CNS WBC ≥ 3 cells and positive cytopathology; ^±^ Cytogenetics abnormality including 46, XY, der(6), t(6;?)(q21;?), add(9)(p21), del(9)(p13) and 46,XX, add(15)(p11.2); ^π^One patient who had late combined medullary/CNS relapsed disease received high-risk chemotherapy protocol due to the decision to proceed without hematopoietic stem cell transplantation. Another patient who had a diagnosis of T-lymphoblastic lymphoma was treated with the high-risk protocol.

**Table 2:** Pharmacokinetic Profiles of Seven Cases who are Eligible for Calculation after Native *E.coli* Asparaginase 10,000 IU/m^2^ Intramuscular Injection

Case profile^π^	A	B	C	D	E	F	G	Min.	Max.	Mean	GMean	Median	SD	CV
AUC time range	0 to 48	0 to 48	0 to 24	0 to 48	0 to 48	0 to 48	0 to 48	-	-	-	-	-	-	-
T_max_	24	24	24	24	24	24	6	6	24	-	-	24	-	-
C_max_	3.47	3.85	3.53	3.51	4.11	3.02	3.71	3.02	4.11	3.6	3.58	3.53	0.34	9.54
Log AUC	137.14	173.93	71.07	143.29	180.12	131.47	165.59	71.07	180.12	143.23	137.99	143.29	36.94	25.8
Post-48-hr activity	3.22	3.51	-	3.3	3.18	2.77	3.18	2.77	3.51	3.19	-	3.2	0.24	-

Abbreviation: AUC or Log AUC, area under the curve; CV, coefficient of variation; C_max_, maximum concentration; SD, standard deviation; T_max_, time-to-peak activity level; ^π^The pharmacokinetic graph was shown in Figure 1.

**Table 3:** Pharmacokinetic Profiles of Case H after Native *E.coli* Asparaginase 10,000 IU/m^2^ Intramuscular Injection and Asparaginase-Specific Antibody Test

Case profile	Case H^π^
AUC time range	0 to 48
T_max_ (h)	48
C_max _(IU/mL)	1.06
Log AUC 0 to 48 (IU.h/mL)	22.84
post-48-hour activity	1.06
Asparaginase-specific antibody (ng/ml)	Positive*

Abbreviation: AUC or Log AUC, area under the curve; C_max_, maximum concentration; T_max_, time-to-peak activity level; ^π^The pharmacokinetic graph was shown in Figure 2; *Asparaginase-specific antibody was 1.31 (ng/ml) (2.8 times of control). A value of more than 2.5 times of normal is considered positive. The mean level of another four volunteers (controlled cases) who did not have history of asparaginase administration was 0.48 ng/ml.

## Discussion

Our study demonstrated that six doses of native *E.coli* asparaginase administrated intramuscularly at a dose of 10,000 IU/m^2^ elucidated sufficient asparaginase effectiveness with a post-48 hour activity level >0.1IU/ml in all 7 cases We had assumed that the activity level might be above the cut-off throughout the given period at 12 days or greater. However, this study did not show evidence of a latency of asparaginase activity after 48 hours (or at 14 days or 21 days or end of induction) which was reported in previous literature at different time points. Due to a small study population, interpretation of our results should be restricted.

This pharmacokinetic study revealed an identical mean time to reach maximum concentration (T_max_) which presented at 24 hours (between 6-48 hours) as previous studies. However, the mean peak serum concentration (C_max_)±SD was 3.60±0.34 IU/ml (range 3.02-4.11 IU/ml) which was 1.5-3.7 times greater than other reports (Riccardi et al., 1981; Avramis et al., 2002).

The AUC ,which represents the total drug exposure across time, was calculated on therapeutic drug monitoring, the possibility of dose adjustment within the reference range apart from the activity level as same as carboplatin and busulfan. Furthermore, it was performed for the calculation of elimination factors. However, no AUC_0-48h_ referable values were purposed for native *E.coli* asparaginase to adjust. Further studies are required.

We described one case of a relapsed patient who had a lower level of activity compared with the other 7 cases in our study. The highest activity was only 1.06 IU/ml. This case had also reported asparaginase clinical hyperreactivity as urticaria. The activity showed clinical correlation with a lower level than usual implying that antibody production might have contributed to this result. This patient was studied during the DI phase where it had been reported to develop antibodies more than during induction time (Avramis et al., 2002). The duration of activity above 0.1 IU/ml for this case is still unknown.

Native *E.coli* asparaginase offers few drug interactions. No reports of other combination chemotherapies as vincristine, doxorubicin, intrathecal methotrexate, or systemic corticosteroid will affect asparaginase activity, although asparaginase may affect certain drugs (Lexicomp Online Database [database on the Internet], 2021). Its molecular weight remains unchanged and no other metabolized products can be found after ex-vivo incubation. It is also not present in urine. The elimination mechanism still be unclear but thought to be driven by the reticuloendothelial system (Pinheiro and Boos, 2004; Brueck et al., 1989).

Many experts have documented the benefit of routinely measuring asparaginase activity in all cases receiving the ALL protocol as the standard of care, because silent inactivation may cause the drug to fall below the cut-off values without clinical notice (van der Sluis et al., 2016). Some of measurement protocols recommend only day 14 after the first dose. Asparaginase activity greater than 0.1 IU/ml indicates medical effectiveness. These recommendations were written for the native *E.coli* asparaginase three-time a week schedule where 72 hours post-dose activity occurs post-14 days of administration. The frequency of monitoring is recommended on the first post-14-day-period in every re-introduction of the drug. This recommendation is based on the native *E.coli* asparaginase-based protocol having a greater benefit to carry out monitoring due to the higher likelihood to induce the antibody production compared to other treatments. When clinical allergy developed or antibodies were detected correlation with subtherapeutic pharmacokinetics study/single-time point level, continuing the same formula caused the ALL treatment to become ineffective. Hence, it is inevitable to switch to a different form of asparaginase. If the clinical allergy is mild or doubtful or has multiple other suspected drugs to cause reaction during the same period, the asparaginase activity with or without the antibody check is also indicated (van der Sluis et al., 2016). Premedication with a steroid or anti-histamine can reduce the allergic symptoms, but no data supports an increase in asparaginase activity or effectiveness. The efficacy of pegylated asparaginase (sufficient activity) after a change from native *E.coli* asparaginase due to antibody production was 77-80% (Asselin, 2011). Switching to pegylated asparaginase should be carefully performed in this situation and clinicians may need to recheck the level during the next 7 and 14 days. The alternative is to switch to Erwinia asparaginase because there is no cross-reactivity especially in patients who have had severe clinical hypersensitivity. 

In practice, only one or two time-point activity is recommended rather than full pharmacokinetic panel. The current available data of correlation between activity and outcome supported monitoring on post 48-hour for native form (Cecconello et al., 2018), post-14 day for pegylated form (Wetzler et al., 2007). No available study of correlation between outcome of Erwinia form and monitoring was varies between study ranged 24,48,72-hour post administration (Salzer et al., 2013; Albertsen et al., 2001; van der Sluis et al., 2016). However, some recommended to monitor at post-48 hour (Brueck et al., 1989). For multiple injection, keeping level above 0.1 IU/ml before the next dose is mandatory. Asparaginase antibodies are still not routinely recommended to monitor in practice (van der Sluis et al., 2016). Although, many studies performed antibody test at multiple various times after asparaginase administration (Dinndorf et al., 2007; Avramis et al., 2002).

The efficacy of pegylated asparaginase has been well established and is the primary reason for its use as the current first-line formula in ALL treatment protocol. In developing countries such as Thailand, however, the national protocol still requires only native *E.coli* asparaginase because pegylated asparaginase is rarely available and affordable. However, we are concerned since some patients may gain greater benefit if they receive pegylated asparaginase, especially cases involving relapsed patients or those at high risk for relapse or patients who have the potential to develop antibody (genetics polymorphism risks).

Native *E.coli* asparaginase administrated intramuscularly at a dose of 10,000 IU/m^2^ 6 doses every other day over a period of 12 days provides a compelling pharmacokinetic effect compared to previous studies. Post-48-hour activity proved medical effectiveness to nearly entire cases. However, only one case developed asparaginase-specific antibody result in the extremely low plasma asparaginase activity. This special condition should have more concern. The asparaginase activity and/or antibody testing is recommended for all cases especially in a relapsed patients, history of high accumulative dose of asparaginase or suspected allergic reaction to the asparaginase. The low asparaginase activity may notice from clinical hypersensitivity development, direct pharmacokinetic-proved low activity, or asparaginase antibody positive status. In this setting, switching to an alternative form of asparaginase may benefit to maintain leukemia treatment efficacy.

## Author Contribution Statement

PC, TT, and PT designed and reviewed the results. AS and PA performed the laboratory studies. SL, HP, KC, DS and PS involved in patients’ care and collected the patient’s data. PC and PT wrote and revised the manuscript. All authors read and approved the final version of the manuscript.
